# Seawater Mixed with One Part Alkali Activated Material: An Environmental and Cost Evaluation

**DOI:** 10.3390/ma17164113

**Published:** 2024-08-20

**Authors:** Xiaoyu Li, Huiyang Liu, Jinxin Xing, Min Gan, Zhiyun Ji, Xiaohui Fan, Zengqing Sun

**Affiliations:** 1 School of Minerals Processing & Bioengineering, Central South University, Changsha 410083, China; 2 Engineering Research Centre of Ministry of Education for Carbon Emission Reduction in Metal Resource Exploitation and Utilization, Changsha 410083, China

**Keywords:** alkali activated material, life cycle assessment, CO_2_ emissions, cost

## Abstract

Concrete production is associated with extensive energy consumption and significant CO_2_ emissions. In addition, tremendous amounts of freshwater are used as a mixing agent. Urgency is increasing to develop sustainable cementitious materials and promote freshwater-saving strategies. An environmentally friendly alternative binder, seawater mixed with one part alkali activated material, is studied. In this work, a cradle-to-gate life cycle assessment was applied to study the equivalent CO_2_ emission and cost properties of the clinker-free binder. The seawater mixed mortar possesses comparable mechanical properties to Portland cement, with 3 d flexural and compressive strengths of 5.3 MPa and 25.2 MPa. In addition, the mortar developed in this work is of similar cost as commercial cement, but reduces CO_2_ emissions by 44.8%.

## 1. Introduction

Cement concrete is the second most used commodity and the most used construction material in the world [1]. Meanwhile, the concrete industry is suffering from increasing environmental and ecological burdens. For example, 8% of worldwide CO_2_ emissions are caused by the production of cement, which is the binding phase that provides strength to concrete [2]. Urgency is increasing to reduce the CO_2_ emissions of cement or develop alternative construction materials. Practices have been made in utilizing industrial by-products (fly ash (FA), ground granulated blast furnace slag (GGBS), silica fume (SF), etc.) as supplementary cementitious materials (SCMs). McLellan et al. [3] stated that a reduction in CO_2_ emissions from 1 t CO_2_/t to 760–860 kg CO_2_/t can be achieved by blending 15–30% SCMs. Limestone blended calcined clay cement (LC^3^) is another trend [4,5]. Low-grade clays are highly efficiently used in this process without capital modification to the existing cement plants. In addition, the LC^3^ can reduce CO_2_ emissions by up to 30% compared with ordinary Portland cement (OPC) [6].

Another choice is developing clinker-free binders, with alkali-activated materials (AAMs) as a representative. The most used solid materials for AAM production are FA, GGBS, metakaolin (MK), etc. and sodium hydroxide and sodium silicate are the most frequently used activators [2,7]. As documented, this history starts from the 1900s, when Kühl patented findings based on a mixture of alkaline components with GGBS. In the 1940s, Purdon conducted detailed investigation and commercialized the products in Belgium. Glukhovsky, former Soviet Union scientist, developed binders based on the alkali activation of low-calcium aluminosilicates [7]. The obtained binders were successfully used in infrastructure and domestic construction. In the 1970s, French scientist Joseph Davidovits patented several alkali-activated aluminosilicates with the terminology ‘Geopolymer’ [8]. Geopolymers are normally regarded as a subclass of AAMs, which refers to the low-calcium or calcium-free group [7]. Extensive work has been conducted on the reaction and properties of geopolymer. Studies have shown that AAMs possess excellent engineering properties, such as rapid hardening, high early and long-term strength, chemical resistance, dimensional stability in service, strong adhesion, etc. [9]. Moreover, AAM production is characterized as low CO_2_ emission because it does away with the high temperature calcination process [2,10]. A tremendous amount of CO_2_ is released during the calcination of carbonate feedstocks together with the grinding process. The equivalent CO_2_ emission is approximately 0.85 t for the production of 1 ton of cement. In comparison, AAMs are estimated to save up to 97% of CO_2_ emissions [2]. 

Benefiting from their superior properties, AAMs possess the possibility of being used in immobilizing hazardous/nuclear wastes and high-volume infrastructure construction. To date, success has been achieved in immobilizing artificial wastes comprising lead (Pb), zinc (Zn) and chromium (Cr(VI)) [11,12]. Chemical entrapment is supposed as one of the solidifying mechanisms. Studies have demonstrated that arsenic (As) is more likely to be associated with iron-rich areas from FA [13]. In addition, the small radius of copper (Cu) ions decreases the tendency of being trapped in nano-pore networks. Blackford et al. [14] reported that cesium (Cs) can be chemically bound in the geopolymer structure as a charge-balancing cation. Hence, better immobilization effects than OPC matrices have been found. Similar results were reported by Li et al. [15]. Apart from these laboratory-level investigations, actual nuclear waste immobilization using geopolymers has been accepted by the Slovak Nuclear Authority [16]. In addition to the aforementioned engineering practices of AAMs, a four-story building was constructed using geopolymer concrete floors in Queensland in 2013. Additionally, one year later, the Brisbane West Wellcamp Airport (BWWA) was opened for business, for which approximately 40,000 m^3^ of geopolymer concrete was used. All these demonstrate that AAMs, including geopolymers, are promising alternative binders.

Freshwater consumption is another problem associated with concrete production. Water withdrawal and consumption occur throughout every major production phase of concrete, including the manufacture of cement and additives, the quarrying and crushing of aggregates, mixing and batching, etc. [1]. According to Miller et al. [1], approximately 1.66 × 10^10^ m^3^ of water is consumed for concrete production every year. Water scarcity is one of the greatest risks all over the world. It is predicted that 75% of water demand from concrete production in 2050 will take place in regions that are most likely to suffer water shortage. Using seawater as a replacement for concrete mixing has been investigated. Although the high chloride content may enhance the corrosion potential of steel reinforcing, positive findings have been obtained from seawater-mixed concrete structures [17]. In addition, steel corrosion can be avoided when using non-corrosive fiber-reinforced polymer (FRP). The FRP-reinforced concrete is reported to possess properties of excellent durability under seawater exposure and significant long-term cost savings [18,19,20]. In our previous work, the micro- and macro-properties of seawater mixed with one part AAM were studied [21]. The obtained materials achieved excellent mechanical properties and only very slight corrosion traces were found on the surface of steel reinforcement after one year. Similarly, AAMs containing seawater and/or sea sand were produced by Yang [22] and Jun [23], respectively. The incorporation of seawater led to the intercalation of chloride ions with layered double hydroxides and the development of early strength [23]. A dense structure was detected with reduced dimensions and a reduced number of micro-cracks in the interfacial transition zone. All these increase confidence in the production of seawater-mixed AAM concrete.

The application of a new material is not only determined by its engineering properties, but also the cost and environment credits. As part of systematic study, this work is designed to characterize the environment and cost properties of seawater mixed with one part AAM mortar. An analysis was conducted using a cradle-to-gate (from resource extraction to products) life cycle assessment (LCA). Comparison with OPC mortar of the same compressive strength grade is included to evaluate whether the AAM possesses environmental and commercial advantages.

## 2. Materials and Methods

### 2.1. Materials

The solid precursors used in this work are FA and GGBS; both are commercially available. Portland cement, CEM I 42.5 N, was selected as a reference. The chemical compositions of FA and GGBS were characterized using X-ray fluorescence (XRF). As shown in Table 1, the FA is mainly composed of SiO_2_ and Al_2_O_3_, with the CaO content being 4.85%. The densities of FA and GGBS are 2.03 g/cm^3^ and 2.94 g/cm^3^, respectively. The surface area was detected using a Blaine air permeability test, which is 3250 cm^2^/g for fly ash and 4127 cm^2^/g for GGBS.

Though not shown here, the mineralogical compositions of source materials were documented using powder X-ray diffraction (XRD) and the results were quantitatively analyzed. GGBS and FA comprise mainly amorphous phases, with the amounts being 73.0% and 94.9%, respectively.

Alkaline activators for AAM synthesis are sodium hydroxide, sodium silicate and sodium carbonate, all solid phase. Sodium silicate possesses an original SiO_2_/Na_2_O molar ratio of 3.5. The mix proportion of each component is listed in Table 2. The mixture was determined based on extensive pre-tests. The seawater used was from Zhoushan, East China Sea. The content of major elements is shown in Table 3. A water-to-binder ratio of 0.45 was applied for the AAM and OPC mixture.

### 2.2. Sample Preparation

Mortar samples were prepared using AAM and OPC, respectively. For the AAM, solid precursors and activators were weighted and mixed in a planet mixer for 3 min. The seawater was then added into the mixer and mixed for 2 min. Standard sand was adopted for mortar production. The mass ratio of sand-to-binder is 3 for all mixtures. After adding sand, another 3 min of mixing was conducted to obtain homogeneous mortar. The fresh mortar was then casted into 40 × 40 × 160 mm^3^ molds and vibrated for 2 min to remove air bubbles. The molds together with casted mortar were covered with a glass plate and stored in a curing box. The temperature and relative humidity (RH) inside the curing box were 20 °C and 100%. Samples were demolded after 24 h, wrapped with PE foil and then further cured at 20 °C, 65% RH until measurement.

### 2.3. Strength Measurement

The compressive and flexural strengths of seawater mixed with one part AAM and reference OPC mortars were documented. Strength measurement was conducted after 3, 7, 14 and 28 days of curing. The tested sample was first visually checked to make sure it was unbroken. The flexural strength measurement was performed using the three-point loading method, with the distance between two supports being 100 mm. A load was applied at the mid span at a rate of 0.05 kN/s. For the flexural strength, three samples were tested. The resultant six halves were used for the compressive strength measurement. The force rate for compressive strength measurement was 2.4 kN/s.

### 2.4. XRD Measurement and Data Analysis

As aforementioned, the mineral composition of source materials was measured using the XRD method. The reaction products of seawater mixed with one part AAM were also characterized. AAM paste was produced and cured under the same procedure as mortar production. After 28 d, the paste was crushed to less than 1 mm. The obtained particles were immersed in isopropanol for 15 min. The mixture was then filtered, rinsed with diethyl ether, dried at 40 °C, and ground to <63 μm. More details can be found in [24,25].

The powder XRD measurement was conducted using an X’Pert Pro Diffractometer (Panalytical, Almelo, The Netherlands). Theta-theta geometry was adopted over the 2θ range of 5–60°. Tube operating conditions of 40 kV and 40 mA were applied to generate the CuKα X-rays. The measured data were analyzed using the software HighScore Plus. Details about the data analysis are reported in our previous work [24,25].

### 2.5. Equivalent CO_2_ Emission

LCA was applied in this work. The main steps are fully described as follows.

#### 2.5.1. Goal and Scope

The investigation objective and designed implementation are covered in the ‘Goal and scope’ step, which describes the evaluated part, accuracy degree and functional unit. In this work, a cradle-to-gate study of seawater mixed with one part AAM was conducted. Apart from evaluating the potential environmental impacts of seawater mixed with one part AAM, a comprehensive comparison with OPC mortar of the same compressive strength grade was also conducted to quantify the pros and cons of AAM produced in this work. 

**Figure 1:**
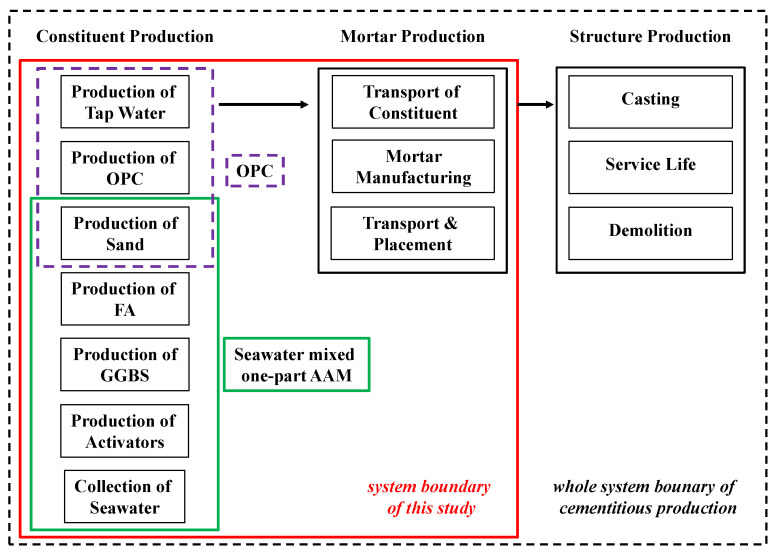
Schematic boundary of this work.

Figure 1 depicts the system boundary of this study, which includes all the associated environmental footprints during the production of solid precursors and activators, production and transport of AAM mortars, etc. The physicochemical properties of solid precursors and mix design significantly influence the reaction kinetics and compressive strength development of the resultant product [26]. In many cases, elevated-temperature curing is necessary. Benefiting from a well-designed formula, excellent engineering properties can be achieved by AAM produced in this work even under ambient curing procedures. Therefore, thermal curing is not included in this work.

The functional unit of this work is 1 m^3^ AAM or OPC mortar, in which the solid binder, water and sand are included. As will be shown below, the excellent mechanical properties of AAM synthesized in this work make it suitable for multiple engineering applications, such as beams, columns, bridges, houses, etc. The work conducted here can not only be extended to the LCA of specific structural products but also to be compared with studies of other construction materials. In addition, the commercially available characteristics of all source materials used in this work make it easy adapt from a laboratory-scale study to industrial scale.

#### 2.5.2. Inventory Analysis

An inventory analysis shows the inputs and outputs of a studied product or system, which are essential for the following impact assessment and interpretation phase [2,7,10]. The primary input data were collected from the Ecoinvent database and the literature [27,28,29,30]. These data have been widely accepted by various researchers and institutions. It is common sense that both GGBS and FA are industrial by-products rather than wastes and both are extensively used as SCMs in concrete production. For example, the yearly production of GGBS is fully used in several European countries. Thus, the allocation of each environmental impact parameter on both materials can be performed based on mass or market price. Studies were conducted to compare the influence of allocation process [27,29]. Results show that no specific allocation method is fully adequate. Generally, allocation based on economic values is more widely used since it represents the reality of industrial processes better. This was also the adopted allocation method of this work.

The alkaline activators used in this work, including sodium hydroxide, sodium silicate and sodium carbonate, were in solid form. The sodium hydroxide was electrolytically produced from chloride salts. Rock salt and sand are the main raw materials for the production of sodium silicate. Sodium carbonate is produced by the Solvay process from sodium chloride and limestone. The inventory data for alkaline activators have been well estimated [31,32,33]. In a conventional AAM production, alkali solutions are used. The alkaline solution is commercially purchased or produced by dissolving solid activator in water. In comparison, the one-part synthesis can avoid the impracticalities of transporting and dealing with large amounts of corrosive alkaline solutions. The contribution introduced by water is normally excluded due to its extremely low environmental footprint. Meanwhile, the effect of water was taken into consideration in this work for a more decent analysis. In the construction materials industry, the environmental inventory of Portland cement and standard sand have been extensively studied based on surveys and statistics [27,28,29,30,31,32]. 

#### 2.5.3. Calculation and Interpretation

Based on the accumulated inventory data, the equivalent CO_2_ emission was calculated using the software Open LCA 2.0. The significant impacts and significant unit processes in the system were identified in interpretation. Conclusions based on the comparison of seawater mixed with one part AAM and Portland cement are given. The benefits of using seawater for the production of AAM with excellent corrosion resistance are included.

### 2.6. Cost Evaluation

The cradle-to-gate cost of seawater mixed with one part AAM was evaluated in a similar way as the equivalent CO_2_ emissions, except for the inventory data. The inventories of cost were based on a literature review of published values and preliminary marketing research [3]. Accumulated data are listed in Table 4.

The cost of a product to the end users is significantly influenced by the distance and mode of transport. Cement, fly ash, slag, chemicals, etc., are localized commodities due to the wide distribution of power plants, steelworks and chemical plants in China. Long-distance transport is rare. In addition, when focusing on a specific city, these factories are normally located in the same industrial park. All these indicate that the corresponding transport mode and distance of cement and AAM are similar. The cost of transport is then not considered in this work, and the most attention is paid to comparing the average cost of all ingredients for AAM and OPC mortars.

## 3. Results and Discussion

### 3.1. Flexural and Compressive Strengths

**Figure 2:**
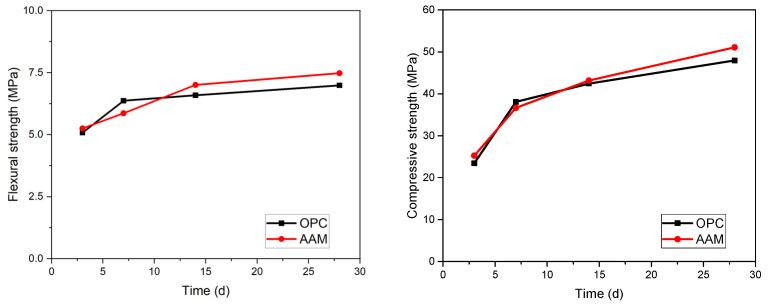
Flexural and compressive strengths of seawater mixed with one part AAM mortar.

Mechanical properties are among the primary factors for the design of cementitious structures. Figure 2 shows the flexural and compressive strength development of seawater mixed with one part AAM, as well as the reference OPC. The 28 d flexural and compressive strengths of AAM and OPC are of the same strength grade, demonstrating the selection of OPC is proper. The flexural and compressive strengths satisfy most structure application scenarios. Slightly higher flexural and compressive strengths of AAM than its OPC counterpart can be seen from Figure 1. In addition, the AAM possesses higher early age flexural and compressive strengths property, with the 3 d flexural and compressive strengths being 5.3 MPa and 25.2 MPa, respectively. This can be assigned to the extensive alkali activation process and the fast formation of flexural and compressive strengths giving phases [24,25]. The high early-age flexural and compressive property is essentially for cementitious materials. For example, when used for pavement construction, this contributes to minimizing traffic control and the early opening of traffic. 

Numerous studies have been reported the flexural and compressive strengths of AAM with different mix designs [2,10]. The flexural and compressive strengths of AAM produced in this work are better than those in many of the reported studies, suggesting the advantage of proportion. It should also be mentioned that the AAM produced in this work is ambiently cured. This is not only energy saving because no heat treatment is required, but also user friendly, especially for in situ application. 

### 3.2. Reaction Product

**Figure 3:**
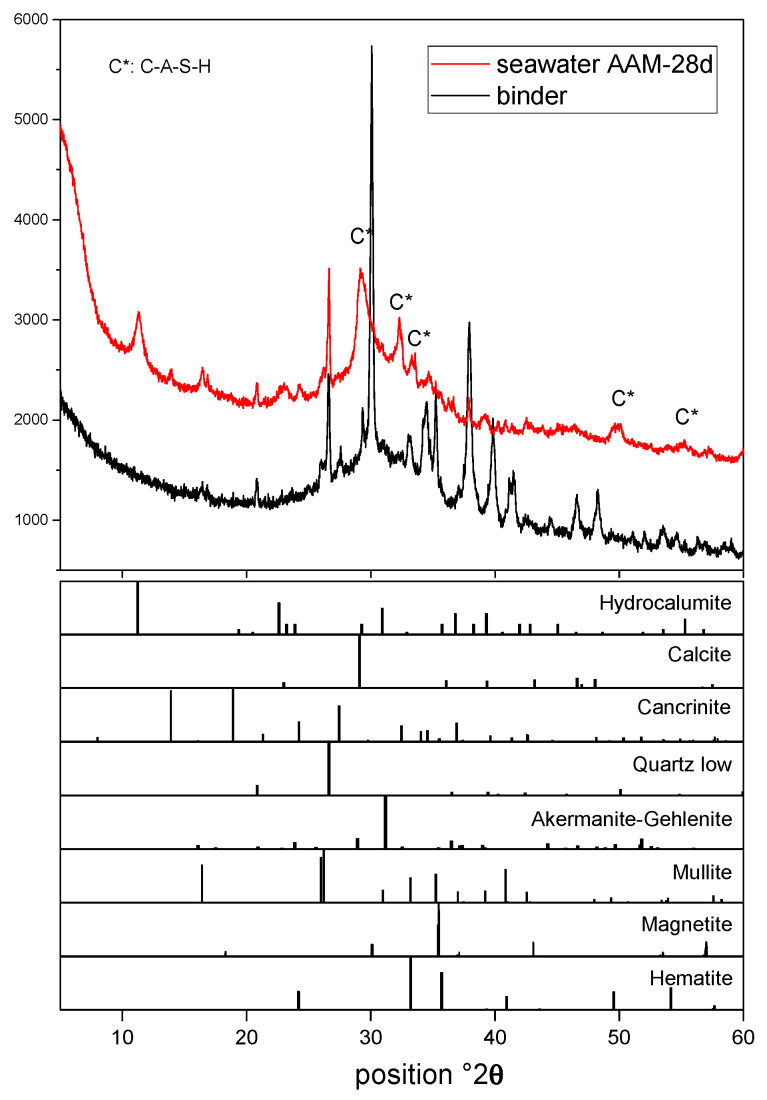
XRD pattern of seawater mixed with one part AAM after 28 d.

The reaction products of AAM were measured by XRD and the results are shown in Figure 3. Compared with the initial pattern of the binder, the reflection peaks of the activator disappeared. When coming into contact with water, the sodium hydroxide hydrolyses promptly and initiates alkaline conditions for the dissolution of sodium silicate. The network breakdown of solid source materials takes place simultaneously. Then the gelation and condensation processes of the dissolved ions are similar to conventional two-part AAMs. The mineral phases contained by solid precursors remain almost unchanged after 28 days, except the anhydrite. The dissolution of anhydrite during the alkali activation process has been reported in our previous studies [24,25].

New reflection peaks can be seen in Figure 3 indicating the formation of crystals. The humps around 25–35° 2θ can be assigned to the calcium (alumino)silicate hydrate type gel (C-A-S-H) [7,10]. The C-A-S-H is the main reaction product and responsible for the engineering properties of AAM. Regarded as aluminum-substituted C-S-H, the substations of aluminum mainly occur in the bridging tetrahedral sites. Puertas et al. [34] conducted experimental and computational studies on C-A-S-H gel; the findings show that it is much more densely packed than C-S-H.

Peaks around 14°, 19°, 24° and 27° 2θ can be assigned to the formation of cancrinite. Cancrinite is a zeolite belonging to the ABC-6 family. According to Oh et al. [35], the basic structure layer of cancrinite consists six member rings of Al- and Si-tetrahedra. The formation of cancrinite has been reported in several studies [24,35].

The reflection peaks of hydrocalumite were detected. Hydrocalumite, with an AFm-like structure, is typical double hydroxides (LDHs) in AAM [27,36]. Due to the substitution of divalent ions by trivalent ones, the LDHs is normally a positively charged layer structure [37]. Additionally, LDHs are capable of interlayer anion exchange. A closer check of the XRD pattern and comparison with the published literature indicates that the hydrocalumite formed in this work is chloride bearing. In the construction industry, ‘Friedel’s salt’ is a term applied to describe chloride-bearing AFm-like phases. Friedel’s salt is important for retaining chloride within a cementitious matrix [38,39]. The detection of chloride-bearing hydrocalumite suggests that the AAM possesses chloride-binding properties, which might be beneficial in protecting steel reinforcement from chloride corrosion. 

### 3.3. Equivalent CO_2_ Emissions

**Figure 4:**
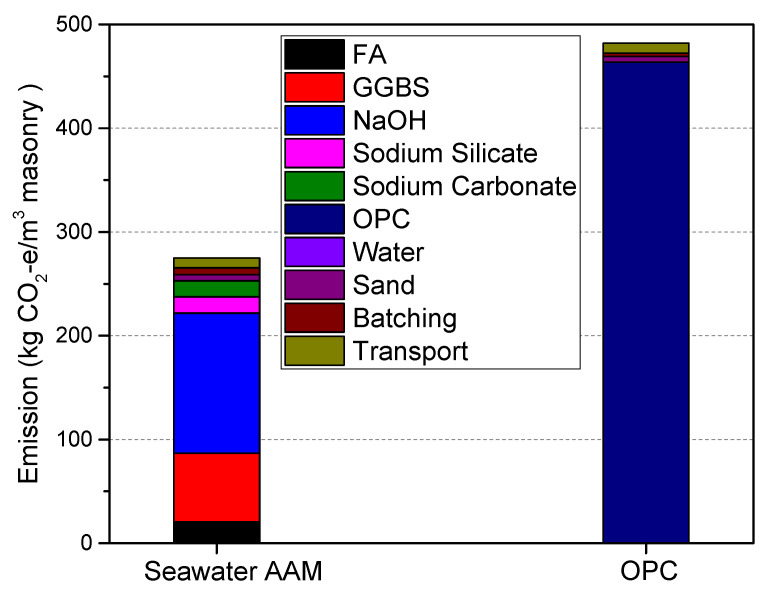
Equivalent CO_2_ emissions of seawater mixed with one part AAM in comparison with OPC mortar.

The equivalent CO_2_ emissions of seawater mixed with one part AAM mortar are shown in Figure 4, compared with OPC mortar. As discussed above, both samples possess similar mechanical strength, indicating that the comparison of life cycle equivalent CO_2_ emissions is meaningful. The equivalent CO_2_ emissions of seawater mixed with one part AAM are 258.9 kg/m^3^. The main contributors are NaOH, GGBS, FA, sodium silicate and sodium carbonate, which account for 52.2%, 25.6%, 8.0%, 6.1% and 5.9% of total CO_2_ emissions, respectively. The high CO_2_ emissions generated from NaOH should be assigned to its production process. NaOH is generally produced by the electrolysis of sodium chloride solution via mercury cells, membrane cells or diaphragm cells. In 2018, sodium hydroxide production in China was over 34 million tons, over 85% of which was produced by the membrane cell technique. Some other obsolete technology is still used in China [31]. A life cycle assessment of NaOH production in China has been conducted by Hong et al. [31]. Electricity efficiency and raw material consumption, accounting for 72% and 19%, are the largest contributors to the overall environmental burdens of NaOH production. Compared with the best available technology in Europe, the NaOH production industry suffers from extensive energy consumption and CO_2_ emissions, high production costs and unbalanced development between NaOH and chlorine products. 

The equivalent CO_2_ emissions of reference for OPC mortar are 469.3 kg/m^3^, of which over 90% is caused by the production of cement. It is well known that cement production is associated with high global warming potential. During cement production, raw materials, such as stone and clay, are calcined to generate clinker in a rotary kiln. Clinker is then ground to fine powder and mixed with gypsum. CO_2_ is released from both fossil fuel combustion (for electricity generation or directly used by cement rotary) and calcium carbonate conversion into oxide form during the calcination process. Though practices have been made in partially replacing clinker by supplementary cementitious materials, the reduction effect on CO_2_ emissions varies [40]. 

Comparing with the reference OPC mortar, equivalent CO_2_ emissions from seawater mixed with one part AAM are reduced by nearly 45%. This once again demonstrates the environmental benefits of AAM. In addition, the equivalent CO_2_ emissions of AAM synthesized in this work are lower than those of other AAMs reported in [3,41,42]. This is partially because of the well mix design and because no thermal treatment is required in this work.

### 3.4. Cost Evaluation

**Figure 5:**
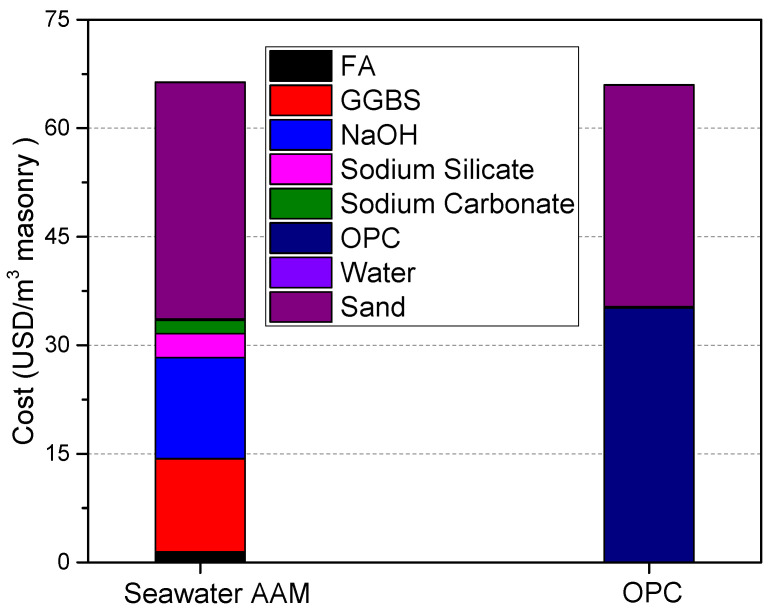
Cost evaluation of seawater mixed with one part AAM and OPC mortar.

As shown in Figure 5, the cost of seawater mixed with one part AAM is almost the same as that of the reference OPC mortar. The cost of OPC mortar is predominantly caused by OPC and sand, with the influence of water being extremely low. Sand makes a similar contribution in the seawater AAM mortar system, which accounts for 47% of the total cost, followed by NaOH, GGBS, sodium silicate, sodium carbonate and FA. The significant influence of sand is assigned to its large consumption amount. Alkali activators, NaOH, sodium silicate and sodium carbonate in total make up 27.5% of the cost.

According to McLellan et al. [3], the cost of AAM is in the range of 7% lower to 39% higher than that of OPC. The calculated results in this work are located in this cost range. It should also be mentioned that the cost is calculated under present pricing structures without taking carbon price into consideration. Nowadays, overall limits and taxes on CO_2_ emissions from energy-intensive industries (production of iron, aluminum, cement, paper, glass, etc.) have been set. These taxes seem to be enhanced by increasingly stringent environmental regulations [43]. As discussed above, AAM reduces CO_2_ emissions by 44.8% over OPC. Thus, when taking this into consideration, the cost of AAM mortar is promising and competitive.

Another benefit of the one-part AAM synthesized in this work is its potential freshwater saving characteristic. According to the United Nations World Water Development Report [44], global water demand will continue to rise at a rate of 20–30% above the current water usage level. The main driven force is the rising demand from industrial and domestic sectors. More seriously, more than 2 billion individuals live under high water stress and 4 billion individuals suffer from serious scarcity for at least one month a year. Concrete production is reported to be responsible for around 9% of the worldwide industrial water consumption [1]. In contrast, seawater covers over 70% of the Earth’s surface and is the most abundant aqueous material on the Earth. Considering the negative environmental impacts of current desalination and the scarcity of freshwater [1,45], studies about using seawater for concrete production are increasing. The influences of seawater on properties of concrete have been reported in [46,47]. In our previous work [21], the micro- and macro- properties of AAM mixed using seawater were studied. Our results favor the possibility of using seawater as an alternative mixing water for AAM concrete production.

## 4. Summary and Conclusions

The equivalent CO_2_ emissions and cost properties of seawater mixed with one part AAM are studied in this work using a cradle-to-gate life cycle assessment method. The AAM mortar possesses excellent mechanical properties. The 28 d flexural and compressive strengths are 7.5 MPa and 51.1 MPa, respectively. Chloride-bearing reaction product was detected in the seawater mixed with one part AAM, suggesting the AAM can contribute to retaining chloride within the matrix. Moreover, the cost of AAM studied in this work is similar to that of commercial Portland cement but reduces CO_2_ emissions by 44.8%. These results suggest that seawater mixed with one part AAM is sustainable and promising.

The authors are highly aware that though the environment and cost were evaluated, much work such as making life cycle inventory more readily available, scoping the assessment, updating impact indicators, etc., should be addressed. In addition, the durability of synthesized AAMs cannot be ignored, since weathering and corrosion will take place during service life. Further attention will be continuously paid to these aspects to promote the applicability of AAMs.

## Figures and Tables

**Table 1:** Chemical composition of FA and GGBS.

Parameter/Compound	FA	GGBS
	wt.%	wt.%
Al_2_O_3_	21.67	10.53
SiO_2_	52.72	40.28
CaO	4.85	34.54
Fe_2_O_3_	9.55	0.39
MgO	1.89	8.63
P_2_O_5_	0.48	0.15
TiO_2_	0.90	0.40
MnO	0.08	1.14
Na_2_O	1.11	0.59
K_2_O	2.27	1.62

**Table 2:** Calculated chemical composition of the seawater mixed with one part AAM paste.

**Compound**	Al_2_O_3_	SiO_2_	CaO	Fe_2_O_3_	MgO	Na_2_O	K_2_O
**Content (wt.%)**	21.67	52.72	4.85	9.55	1.89	1.11	2.27

**Table 3:** Composition of the seawater used in this work, given in g/L.

	Na^+^	K^+^	Ca^2+^	Mg^2+^	Cl^−^	SO_4_^2−^	Br^−^	HCO_3_^−^
Seawater	11.87	0.38	0.39	1.33	20.95	2.97	0.11	0.14

**Table 4:** Market price of raw materials used in this work.

Material	Market Price (USD/t)
FA	7.3–21.8
GGBS	30.2–35.1
NaOH	421.4–501.7
Sodium silicate	316.1–372.7
Sodium carbonate	206.3–247.1
OPC	56.4–71.6
Sand	14.3–22.9
Water	0.52–1.01

## Data Availability

The original contributions presented in the study are included in the article, further inquiries can be directed to the corresponding author.
